# Virulence of *Mycobacterium tuberculosis* Clinical Isolates Is Associated With Sputum Pre-treatment Bacterial Load, Lineage, Survival in Macrophages, and Cytokine Response

**DOI:** 10.3389/fcimb.2018.00417

**Published:** 2018-11-27

**Authors:** Trinh T. B. Tram, Hoang N. Nhung, Srinivasan Vijay, Hoang T. Hai, Do D. A. Thu, Vu T. N. Ha, Tran D. Dinh, Philip M. Ashton, Nguyen T. Hanh, Nguyen H. Phu, Guy E. Thwaites, Nguyen T. T. Thuong

**Affiliations:** ^1^Oxford University Clinical Research Unit, Ho Chi Minh City, Vietnam; ^2^Nuffield Department of Medicine, Centre for Tropical Medicine and Global Health, University of Oxford, Oxford, United Kingdom; ^3^Hospital for Tropical Diseases, Ho Chi Minh City, Vietnam

**Keywords:** *Mycobacterium tuberculosis*, clinical isolates, virulence, sputum, bacterial growth, cytokine response, Beijing lineage

## Abstract

It is uncertain whether differences in *Mycobacterium tuberculosis* (*Mtb)* virulence defined *in vitro* influence clinical tuberculosis pathogenesis, transmission, and mortality. We primarily used a macrophage lysis model to characterize the virulence of *Mtb* isolates collected from 153 Vietnamese adults with pulmonary tuberculosis. The virulence phenotypes were then investigated for their relationship with sputum bacterial load, bacterial lineages, bacterial growth, and cytokine responses in macrophages. Over 6 days of infection, 34 isolates (22.2%) showed low virulence (< 5% macrophages lysed), 46 isolates (30.1%) showed high virulence (≥90% lysis of macrophages), and 73 isolates (47.7%) were of intermediate virulence (5–90% macrophages lysed). Highly virulent isolates were associated with an increased bacterial load in patients' sputum before anti-tuberculosis therapy (*P* = 0.02). Isolate-dependent virulence phenotype was consistent in both THP-1 and human monocyte-derived macrophages. High virulence isolates survived better and replicated in macrophages one hundred fold faster than those with low virulence. Macrophages infected with high virulence isolates produced lower concentrations of TNF-α and IL-6 (*P* = 0.002 and 0.0005, respectively), but higher concentration of IL-1β (*P* = 5.1 × 10^−5^) compared to those infected with low virulence isolates. High virulence was strongly associated with East Asian/Beijing lineage [*P* = 0.002, Odd ratio (OR) = 4.32, 95% confident intervals (CI) 1.68–11.13]. The association between virulence phenotypes, bacterial growth, and proinflammatory cytokines in macrophages suggest the suppression of certain proinflammatory cytokines (TNF-α and IL-6) but not IL-1β allows better intracellular survival of highly virulent *Mtb*. This could result in rapid macrophage lysis and higher bacterial load in sputum of patients infected with high virulence isolates, which may contribute to the pathogenesis and success of the Beijing lineage.

## Introduction

*Mycobacterium tuberculosis* (*Mtb*) is the leading infectious cause of death globally, causing active tuberculosis (TB) in 10.4 million and killing 1.3 million people annually (World Health Organization, 2017). *Mtb* is an intracellular pathogen that requires human disease to replicate and spread. One of the most intriguing aspects of tuberculosis is the wide variation in clinical manifestations, disease severity and outcome, which makes it difficult to diagnose, treat, and control. The variation has been primarily attributed to host factors (Berrington and Hawn, 2007; Thuong et al., 2016), but there is evidence suggesting that differential *Mtb* virulence could also be important (Malik and Godfrey-Faussett, 2005). A better understanding of how virulence varies between *Mtb* strains and genetic determinants of virulence would inform efforts to develop new treatments. This knowledge also would help in appraisal of potential virulence-related antigens, which may contribute to the design of novel antitubercular vaccines.

*Mtb* virulence has been characterized *in vitro* in various macrophage models and *in vivo* using animals (Prozorov et al., 2014). Virulence differences have been defined by bacterial growth in cells or organs, the death of infected cells or animals, and by differences in the histopathology of infected animal tissues (Dormans et al., 2004; Sohn et al., 2009). Highly virulent *Mtb* isolates appear to grow faster (Theus et al., 2005), to cause more lung damage and higher mortality (Manca et al., 2001; Dormans et al., 2004), and to be more efficient at transmission (Marquina-Castillo et al., 2009) than attenuated or low virulence strains. These phenotypes may be driven by a reduced or delayed host proinflammatory cytokine response (Manca et al., 2001; Theus et al., 2005; Coscolla and Gagneux, 2014); although some studies have observed increased virulence correlated with increased TNF-α, IL-6, and IL-1β expression (Park et al., 2006; Krishnan et al., 2011). Thus, it is still unclear how virulent *Mtb* clinical isolates manipulate the host immune response to increase their survival and contribute to disease progression and transmission.

Clinical and epidemiological studies have suggested that East Asian/Beijing strains were likely to progress to active TB disease, be associated with extra-pulmonary TB, multidrug resistance, treatment failure, and relapse (Caws et al., 2008; Thwaites et al., 2008; Parwati et al., 2010). The virulence of East Asian/Beijing strains has been evaluated both *in vitro* and *in vivo* but the results have been inconsistent, demonstrating by a wide range of growth rates, and proinflammatory phenotypes (Theus et al., 2007; Aguilar et al., 2010; Portevin et al., 2011). To date, there have been many publications studying strain/lineage-specific virulence; most of them have been limited to laboratory strains or to a few selected clinical isolates and *Mtb* virulence was often assessed based on either bacterial factors or host immune responses, which may explain the conflicting findings. Moreover, how differences in *Mtb* virulence contribute to infection establishment, dissemination, and disease transmission remains unclear.

To address the limitations of previous studies, we systematically characterized the virulence of *Mtb* isolates collected from a cohort study (*n* = 153) by examining the lysis of infected macrophages. We then investigated the association between the virulence phenotypes and bacterial load in sputum samples from TB patients, bacterial lineages, *Mtb* growth, and host cytokine responses in macrophages. Our hypothesis was that *Mtb* clinical isolates have a wide spectrum of virulence, which is lineage-associated, modulates host immune response, and determines bacterial load in patients with pulmonary tuberculosis.

## Materials and methods

### Bacterial isolates

*Mtb* isolates used in this study were collected from a cohort of participants with pulmonary TB (PTB) and were described previously (Vijay et al., 2017). One hundred and fifty three PTB patients were recruited from two district TB control units (4 and 8) in Ho Chi Minh City (HCMC) in southern Vietnam between January 2015 and October 2016. Patients had clinical symptoms of active PTB, which was confirmed by chest X-rays and sputum positive with acid fast bacilli by Ziehl-Neelsen stain. All were adults and HIV negative.

### Ethics approval statement

All patients recruited to the study were ≥18 years old and written informed consent was obtained from each patient or an accompanying relative if the patient was unable to provide consent independently, which was approved by Ethics Committees. The study protocol and informed consent form were approved by the institutional review boards of the Hospital for Tropical Diseases in Vietnam and the Oxford Tropical Research Ethics Committee in the United Kingdom (Vijay et al., 2017).

The whole blood was obtained from three healthy donors who are Vietnamese volunteers working at Oxford University Clinical Research Unit (OUCRU). The samples were part of an existing study at OUCRU on the influence of host genotype to disease susceptibility phenotype using a healthy cohort of Vietnamese adults. Participants enrolled to this study had consented to donate samples in future studies. These samples were anonymized by subject codes, without names or identifying information.

### Sputum collection and bacteria growth

Sputum samples were collected from 153 PTB patients before the start of anti-TB therapy. The samples were decontaminated by N-acetyl-L-cysteine and 2% NaOH, inoculated in Mycobacteria growth indicator tubes and then on Lowenstein–Jensen (LJ) medium (Becton Dickinson (BD) at 37°C for 3–4 weeks to isolate *Mtb*. Single colonies were sub-cultured in 7H9 liquid medium and stored as glycerol stocks for further experiments. To prepare cultures for infection, *Mtb* isolates and H37Rv were inoculated in 7H9T medium (7H9 broth supplemented with 10% Oleic acid/Albumin/Dextrose/Catalase (OADC) enrichment, and 0.05% Tween 80, BD) at 37°C with shaking. The bacteria were ready for infection experiments when OD_600_ reached 0.5–1.

### Preparation of THP-1 and human monocyte-derived macrophage

THP-1 human monocytic cell line was obtained from ATCC (TIB-202). Cells were cultured in RPMI 1640 (Sigma) supplemented with 10% heat-inactivated fetal bovine serum (FBS, Sigma), 2 mM L-glutamine (Sigma) and 100 units of penicillin (Sigma). Cells were transferred to either 96-well plates (6 × 10^4^ cells per well) or 24-well plates (2.5 × 10^5^ cells per well) to establish a confluent monolayer. The cells were differentiated with 50 ng/ml phorbol 12-myristate 13-acetate (PMA, Sigma) for 2 days and then incubated in fresh media for at least 12 h before experiments.

To prepare human monocyte-derived macrophages (hMDM), peripheral blood monocyte cells (PBMCs) were separated from 20 ml heparinized whole blood by Lymphoprep (Axis-Shield) gradient centrifugation in accordance with the manufacturer's protocol. PBMCs were plated in cell-culture treated 60 mm × 15 mm petri dishes (Corning) with 6–8 × 10^6^ cells per dish in media without serum, containing RPMI-1640 (Sigma), 2 mM L-glutamine (Sigma), and 100 units of penicillin and streptomycin (Sigma). After 2 h, the non-adhered cells were washed off gently three times by warm phosphate buffered saline (PBS) with 3% FBS. Cells were re-suspended in 2 ml complete media containing 10 ng/ml human macrophage colony-stimulating factor (m-CSF, R&D system). On the following day, cells were seeded in 96-well plate (8 × 10^4^ cells per well). Complete media was changed at day 4, and macrophages were infected with *Mtb* at day 7.

### Macrophage infection and observation of macrophage lysis

Prior to the infection, *Mtb* was bead beaten to remove clumps. Macrophages were infected with *Mtb* in uptake buffer (0.5% BSA, 0.45% dextrose, 0.1% gelatin, 0.01 g CaCl_2_, 0.01 g MgCl_2_) at multiplicity of infection (MOI) of 1. Macrophages were incubated for 4 h, then washed twice and incubated for 6 days. The percentage of macrophage lysis was observed daily using light microscopy by two independent staff and blind sampling. Each experiment condition was triplicated and mean values determined.

### Sputum bacterial load measured by genexpert

The bacterial load in all sputum samples was measured using GeneXpert MTB/RIF assay (Cepheid, Sunnyvale, CA, United States) following the manufacturer's standard operating procedure. Briefly, 2 ml sample reagent was added to 200 μl of decontaminated sputum, vortexed for 30 sec, incubated at room temperature for 10 min, then added to the test cartridge, and finally loaded onto a GeneXpert instrument. Results were reported as cycle threshold (Ct) values, representing the number of PCR cycles required for the signal to reach a detection threshold. The average Ct values of five probes (excluding any delayed values due to rifampicin resistance) was used to estimate bacterial load (Blakemore et al., 2010).

### *Mtb* lineage identification and whole genome sequencing

*Mtb* DNA was extracted from cultures on LJ media by cetyltrimethyl ammonium bromide (CTAB) method. Lineages were identified by large sequence polymorphism (LSP) typing method as described previously (Caws et al., 2008; Vijay et al., 2017). Briefly, all the strains were first screened for either RD105 or RD239 deletions by PCR, as the majority of strains were anticipated to contain these deletions. Isolates bearing RD105 deletion were defined as East-Asia/Beijing genotype while those bearing RD239 deletion were defined as Indo-Oceanic genotype. Isolates without RD105 or RD239 deletions were further tested for Euro-American lineage by using PCR to detect the deletion of 7bp in the *pks* gene. For whole genome sequencing, *Mtb* DNA from each isolate was used to prepare a library using NEBNext Ultra II DNA Library Prep Kit for Illumina (New England BioLabs). The libraries were quantified using KAPA Library Quantification low ROX qPCR mix (Roche) and then sequenced with either 300-cycle NextSeq 500 Mid Output V2, or High Output V2 kits, or 600-cycle MiSeq Reagent V3 Kit (Illumina) with the standard Illumina procedure using Illumina NextSeq or MiSeq sequencers.

### *Mtb* survival in THP-1 measured by colony forming unit assay

*Mtb* was infected to 2.5 × 10^5^ THP-1 cells in 24-well plate at MOI 1. After 4 h of incubation (considered as day 0), all extracellular bacteria were removed gently by washing and intracellular bacteria were harvested. At 6 days after infection, both extracellular bacteria released from macrophage lysed in supernatant and intracellular bacteria in intact cell layer were harvested. Bacteria at day 0 and day 6 were plated on Middlebrook 7H10 agar plates in triplicate, plates were incubated for 3–weeks at 37°C and CFU were counted. The growth index was defined as the number of CFU per ml at day 6 divided by the number of CFU per ml on day 0 (Wong et al., 2007).

### Cytokine measurement

Culture supernatants from uninfected and infected hMDM were harvested at 24 h post-infection and stored at −80°C. TNF-α, IL-6, and IL-1β concentrations were measured from stored supernatants at the same time using an enzyme-linked immunosorbent assay kit according to the manufacturer's instructions (DuoSet ELISA, R&D).

### Statistical and bioinformatic analysis

A linear trend test was used to calculate the differences in *P*-value across all three virulence phenotypes. The *P* ≤ 0.05 indicated an increasing or decreasing of bacterial load, growth, or cytokine response, associated with the increased level of bacterial virulence from low to intermediate to high. The *P*-value was computed using independence_test function of R package coin developed by Hothorn et al. (2008). The frequencies of *Mtb* lineages among different virulence phenotypes were compared by Chi-square test. *P*-values of ≤ 0.05 were considered statistically significant. Statistical analyses were performed using statistical package R v3.3.1 (R Core Team, 2016). Graphs were generated by GraphPad Prism v7.03 (GraphPad Software, San Diego, California, USA). For analysis of whole genome sequencing (WGS), FASTQ data were mapped against the H37Rv reference (NC_000962.3) using BWA mem (Li, 2013), SNPs were called using GATK v3.3.0 (McKenna et al., 2010) in unified genotyper mode. Core genome positions that had a high quality SNP (>90% consensus, coverage >5x, genotype quality >30, mapping quality >30) in at least one strain were extracted using snp-sites (Page et al., 2016) and used as the input for maximum likelihood phylogenetic analysis using IQ-TREE v1.6 (Nguyen et al., 2015) with automatic model selection by ModelFinder (Kalyaanamoorthy et al., 2017). *Mtb* lineage was predicted by Mykrobe (Bradley et al., 2015).

## Results

### Virulence of *Mtb* clinical isolates based on THP-1 cell lysis

**Figure 1:**
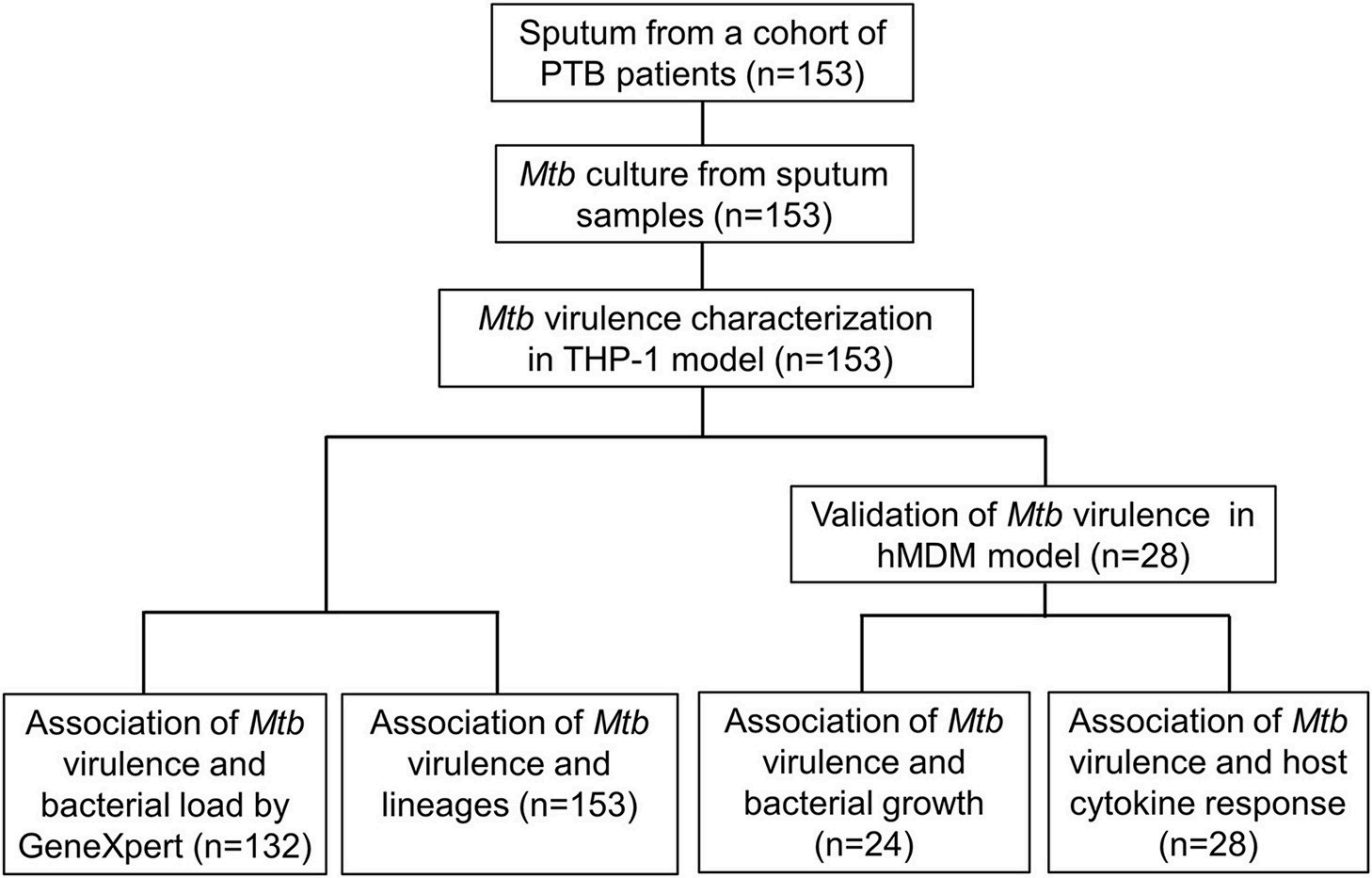
Study design.

**Figure 2:**
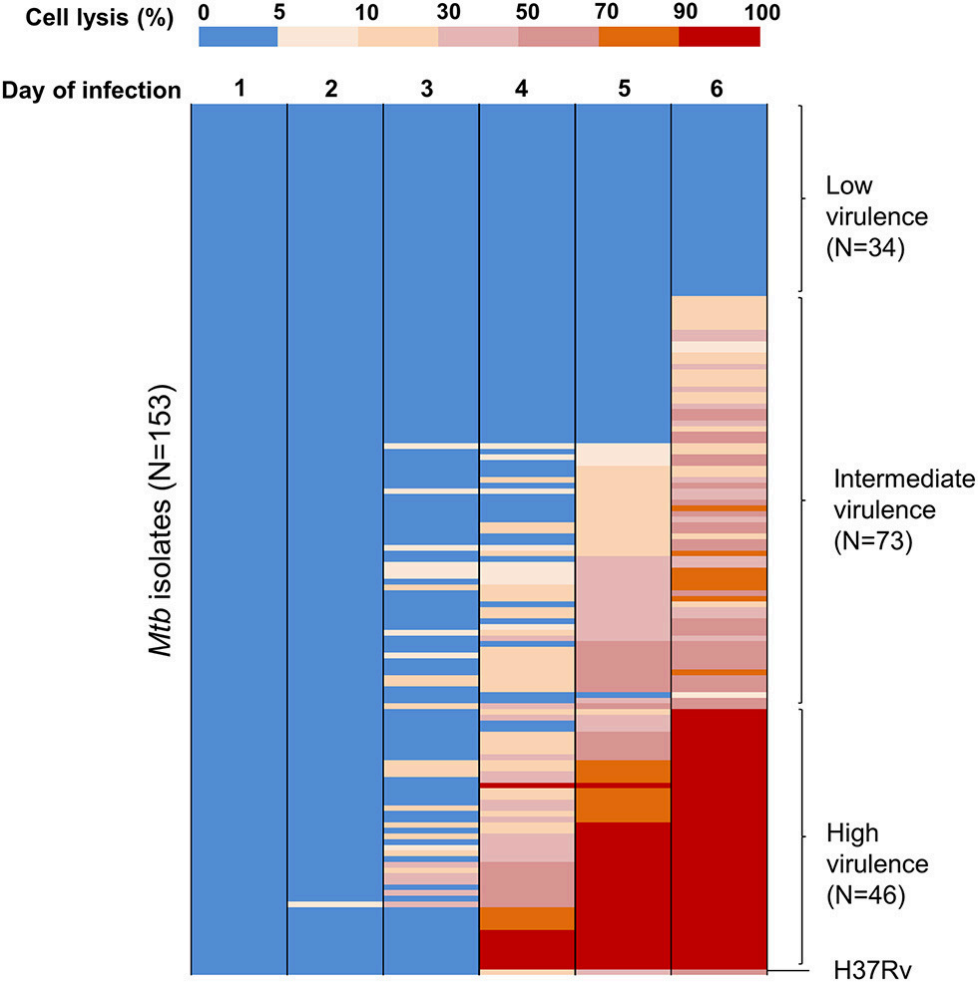
Heat map of THP-1 cell lysis induced by different *Mtb* clinical isolates over 6 days post-infection. THP-1 cells were infected with 153 *Mtb* isolates and lab strain H37Rv at MOI 1. Colors from blue to red represent the proportion of THP-1 lysis, ranging from 0 to 100%. At day 6 of infection, the strains that caused cell lysis up to 90–100% (in red) were clustered as a group of high virulence phenotype, those causing 0–5% (in blue) as low virulence and the remainder as intermediate virulence. The experiments were performed in triplicate and mean values presented.

To assess virulence of *Mtb* strains, THP-1 macrophages were infected with 153 clinical isolates and the lab strain H37Rv (Figure 1). We examined the interaction of macrophages and *Mtb* every day for 10 days after infection. Macrophages were completely lysed (≥90% cell lysis) by a few isolates at day 4 and by half of isolates at day 7. From day 8 onwards uninfected macrophages were unhealthy due to old culture medium. Therefore, we chose day 6 after infection as an optimal time to characterize the virulence phenotype of *Mtb* isolates. The percentage of cells lysed was measured repeatedly for 6 days after infection (Figure 2). During the first 3 days, most of the infected macrophages still adhered and were intact (< 5% cell lysis). On later days, we observed significant variation in the level of cell lysis, ranging from 0 to 100%. For example, on day 4, infected macrophages remained intact in 80 (52.3%) isolates, 5–10% were lysed in 9 isolates, 10–30% in 30 isolates, 30–50% in 14 isolates, 50–70% in 8 isolates, 70–90% in 4 isolates, and completely (≥90%) in 8 isolates (Supplementary Table S1).

We grouped clinical isolates into three different phenotypes of virulence based on macrophage lysis at 6 days after infection: low virulence if < 5% infected cells were lysed, high if ≥90% infected cells were lysed, and intermediate for the remainder. Of the 153 clinical isolates tested, 34 (22.2%) had low virulence, 46 (30.1%) had high virulence, and 73 (47.7%) were intermediate (Figure 2). The lab strain H37Rv exhibited intermediate virulence in this model, causing ~50% macrophage lysis.

### Association between *Mtb* virulence and pre-treatment bacterial load in TB patients

**Figure 3:**
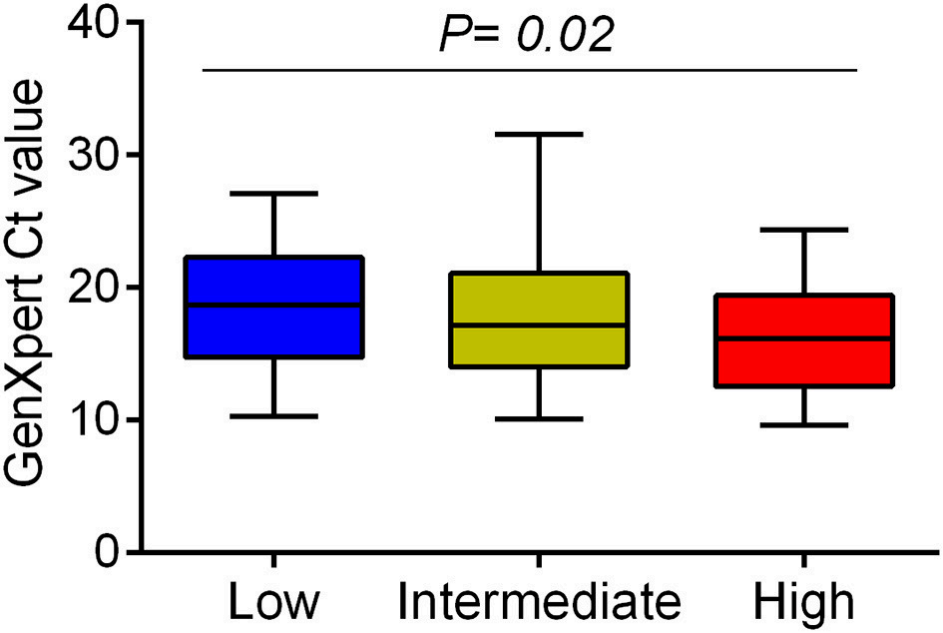
Association between *Mtb* virulence and bacterial load in sputum samples before treatment. *Mtb* load in sputum of 132 PTB patients (29 low, 62 intermediate, and 41 high virulence isolates) was measured by GeneXpert and indicated by Ct values. Bars in plots represent median values. Statistical comparison was made using a linear trend test across the three virulence phenotypes.

We examined whether *Mtb* virulence phenotypes were associated with bacterial load in the sputum of PTB patients before treatment (Figure 1). The linear trend test showed increased *Mtb* virulence was associated with increased bacterial load (*P* = 0.02). The median Ct values of *Mtb* isolates with intermediate and high virulence were 2 and 3 cycles lower, respectively, than those of low virulence isolates, meaning that the numbers of bacilli in sputum were ~4 and 8 times higher for intermediate and high virulence isolates, respectively, than for low virulence (Figure 3).

### Relationship between *Mtb* virulence and east asian/beijing lineage

153 *Mtb* isolates were first genotyped by LSP typing. In line with previous reports (Caws et al., 2008; Thwaites et al., 2008), the three main *Mtb* lineages known in Vietnam were observed in our isolate collection. East Asian/Beijing lineage represented 78/153 (51.0%) of isolates, Indo-Oceanic lineage 50/153 (32.7%), and Euro-American just 18/153 (11.8%). The lineage of a small proportion of isolates (7/153, 4.5%) was undefined due to limitations of the typing method (Caws et al., 2008). To investigate whether virulence is associated with East Asian/Beijing lineage, we combined Indo-Oceanic, Euro-American (including lab strain H37Rv) and the unknown group as non-Beijing lineage to compare with East Asian/Beijing. We found that isolates with high virulence phenotype were likely to belong to East Asian/Beijing lineage rather than non-Beijing lineage (*P* = 0.002, OR = 4.32, 95% confidence interval (CI) 1.68–11.13) (Table 1).

**Table 1:** Association between *Mtb* virulence and lineage.

**Phenotype**	East Asian/Beijing^*^(*N*, %)	Non-Beijing^*^ (*N*, %)	***P***	**OR (95% CI)**
Low (*N* = 34)	11 (14.1)	23 (30.3)	
Intermediate (*N* = 74)	36 (46.2)	38 (50.0)	0.11	1.98 (0.84–4.63)
High (*N* = 46)	31 (39.7)	15 (19.7)	0.002	4.32 (1.68–11.13)

N, number of isolates.

P, P value determined using Chi-square test.

OR (95%CI), odds ratio (95% confidence interval).

* *Lineages were identified by LSP typing, including lab strain H37Rv*.

**Figure 4:**
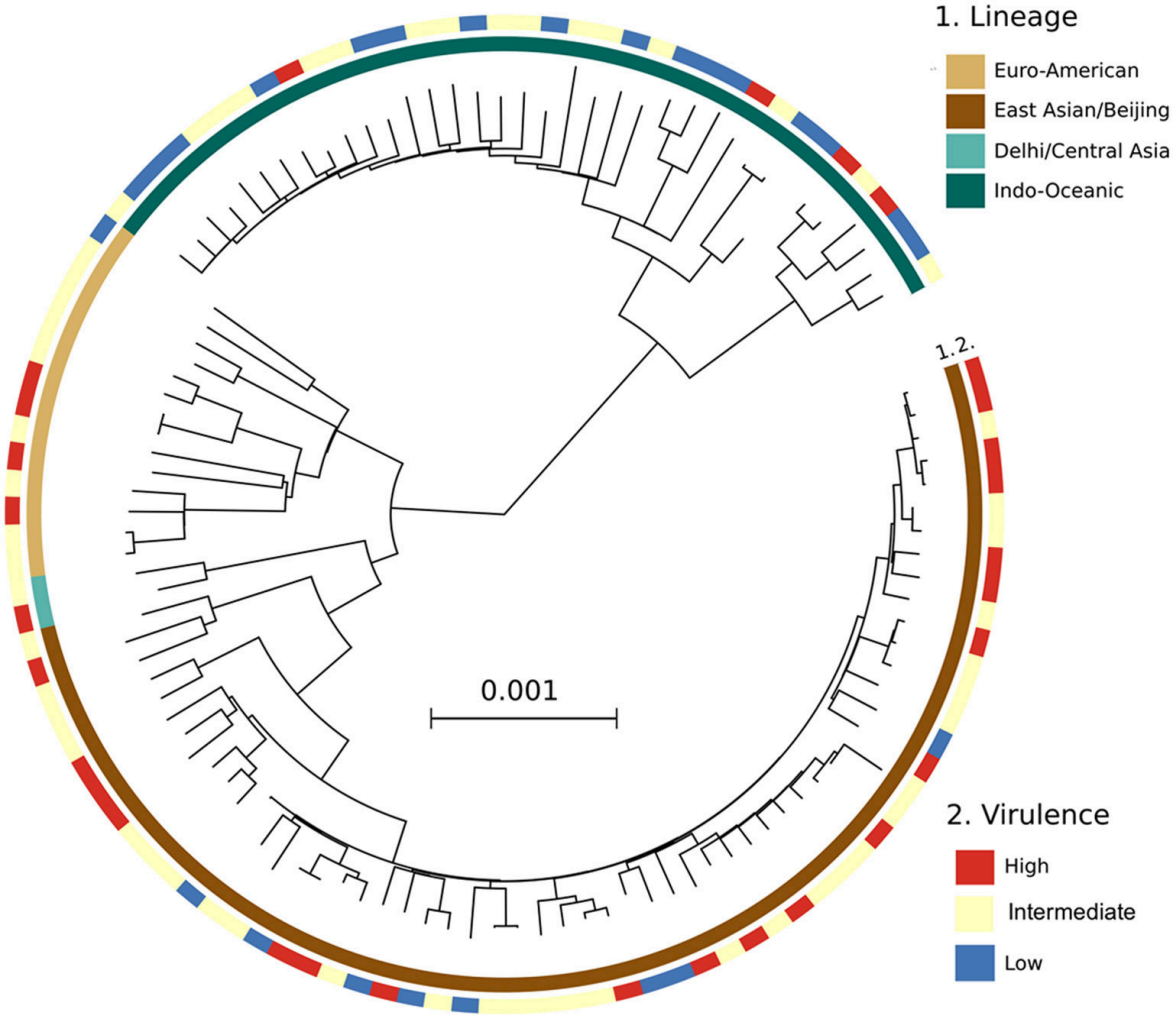
A maximum likelihood phylogenetic analysis of 125 *Mtb* clinical isolates, annotated with their virulence phenotype. Interior and exterior rings of color blocks indicate the different *Mtb* lineages and virulence phenotypes, respectively.

A phylogenetic tree constructed from the whole genome sequencing data of 125/153 *Mtb* isolates (28 were not sequenced due to missing clinical data for these patients) showed similar frequencies of *Mtb* lineages to that from LSP typing. 66/125 (52.8%) isolates belonged to East Asian/Beijing, 40/125 (32%) to Indo-Oceanic, 15/125 (12%) to Euro-American, 2/125 (1.6%) to Delhi/Central Asia lineage, and 2/125 (1.6%) were undefined. Consistently, we observed a high distribution of highly virulent isolates among the East Asian/Beijing lineage (Figure 4).

### Validation of *Mtb* virulence in primary HMDM

**Figure 5:**
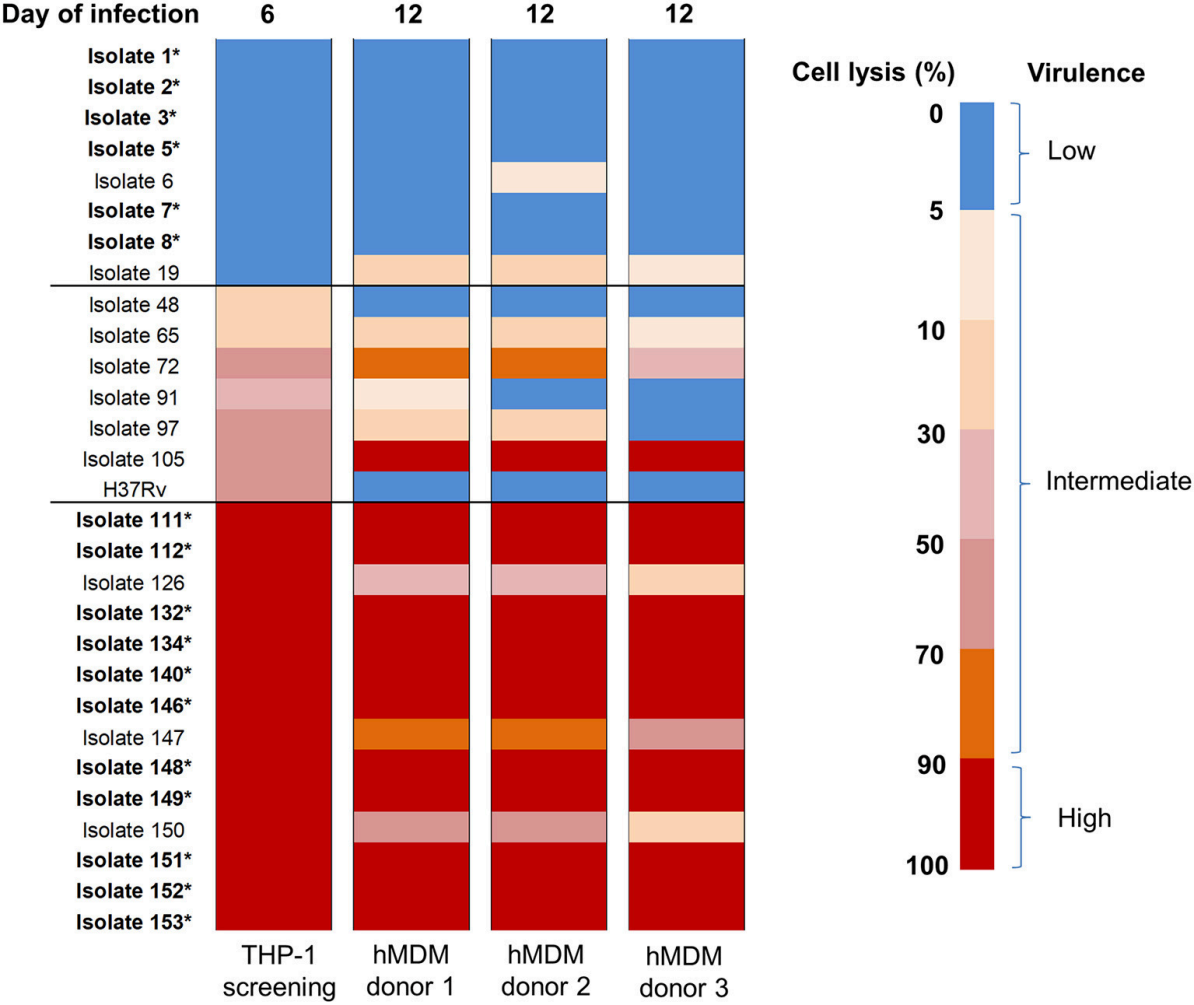
Heat map of hMDM lysis induced by *Mtb* clinical isolates at day 12 post-infection. hMDM from three subjects were infected with 28 *Mtb* isolates and lab strain H37Rv at MOI 1. Colors from blue to red represent the proportion of cell lysis, ranging from 0 to 100%. The virulence phenotype in THP-1 was determined by cell lysis at day 6 while in hMDM at day 12.Strains that caused cell lysis up to 90–100% (in red) were considered as high virulence phenotype, 0–5% (in blue) low virulence and the remainder intermediate virulence. The isolates whose phenotypes were consistent thorough screening in THP-1 cells and hMDM from different subjects were indicated in bold with an asterisk. The experiments were performed in triplicate and mean values presented.

After using THP-1 cell line to characterize the virulence phenotypic diversity among the strains when in the same host environment, we used hMDM from three different donors to validate virulence phenotype and also to investigate the interaction of human macrophages and *Mtb* isolates through host immune response (Figure 1). We randomly selected 28 isolates representative of different phenotypes, comprising 8 strains with low virulence, 14 strains with high, and 6 strains with intermediate virulence, and infected them together with the intermediately virulent H37Rv into hMDM. In comparison to THP-1 cells, the lysis of hMDM was slower, with complete destruction first being observed 12 days after infection. Hence, the virulence phenotype of clinical isolates in hMDM was categorized based on the same criteria as for THP-1 cells (low virulence if < 5% infected cells were lysed, high if ≥90%, intermediate for the remainder) but at day 12 (Figure 5).

Generally there was a similar trend in the virulence phenotype of *Mtb* isolates in the different donors even though host cell lysis was slightly different for 9/29 isolates, with slightly lower levels in donor 3 than in others (Figure 5). The differences could be due to variations in host response. Comparing the two macrophage models, 6/8 (75%) isolates with low virulence in THP-1 exhibited the same phenotype in hMDM while 1/8 (isolate 19, 12.5%) changed to intermediate. Among isolates with high virulence in THP-1, 11/14 (78.6%) remained high across all 3 donors; whereas 3/14 (21.4%) became intermediate. Less consistency was observed with isolates having intermediate virulence. However, overall the results showed that the majority (75%) of isolates conserved their low or high virulence phenotype in both THP-1 and hMDM, with none changing from low to high or vice versa.

### Association between *Mtb* virulence and bacterial growth in THP-1

**Figure 6:**
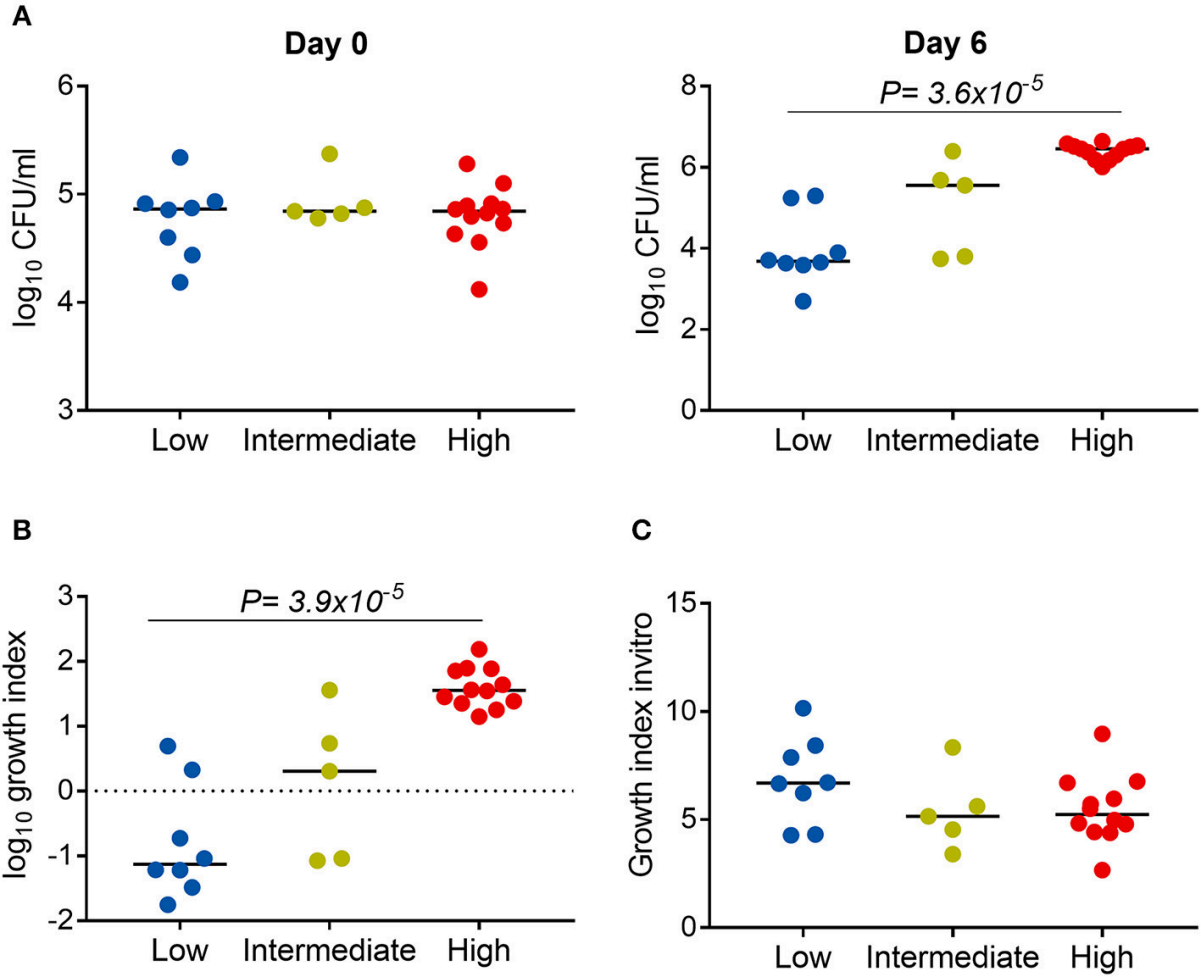
Association between *Mtb* virulence and bacterial growth in THP-1. Twenty four *Mtb* clinical isolates and lab strain H37Rv (with 8 low, 5 intermediate, and 12 high virulence) were used to infect to THP-1 cells at MOI 1 **(A,B)** or cultured in 7H9T liquid media **(C)**. **(A)** Numbers of bacterial colony forming units (CFU) were determined at day 0 and day 6 after infection. **(B)** Change in the number of CFU at day 6 in comparison to day 0. **(C)** Change in the OD_600_ measurement after 20 days of culture in liquid media. Experiments were performed in triplicate and mean values presented. Bars in plots represent median values. Statistical comparisons were made using a linear trend test across the three virulence phenotypes.

We next examined whether the virulence phenotype of *Mtb* clinical isolates was associated with their survival and growth in macrophages (Figure 1). Twenty four clinical isolates and H37Rv, whose virulence phenotypes were validated in both THP-1 and hMDM models, were infected to THP-1 cells at MOI 1; the number of intracellular CFU was determined at day 0 and both intra- and extracellular CFU measured at 6 days after infection. At day 0, there was no difference in the bacillary inoculum of isolates with low, high, and intermediate virulence (Figure 6A). At day 6, greater virulence was associated with an increased number of bacteria (linear trend test, *P* = 3.6 × 10^−5^). Between day 0 and day 6, an almost hundredfold increase in CFU was observed in highly virulent isolates, while in isolates with low virulence the CFU decreased over tenfold (Figure 6B). There was no difference in the growth rate of the strains by virulence phenotypes in 7H9 broth *in vitro* (linear trend test, *P* = 0.12) (Figure 6C). Therefore, the high virulence phenotype as indicated by rapid host cell lysis is likely to be highly associated with their survival and replication ability in THP-1.

### Association between *Mtb* virulence and cytokine concentrations

We hypothesized that the virulence phenotypes of *Mtb* isolates were driven by their ability to modulate host cytokine response. To test this hypothesis, we infected hMDM from three independent donors with each of the 29 *Mtb* isolates described above (including H37Rv) (Figure 1). Cell supernatants were collected after 24 h and concentrations of TNF-α, IL-6, IL-1β were determined.

**Figure 7:**
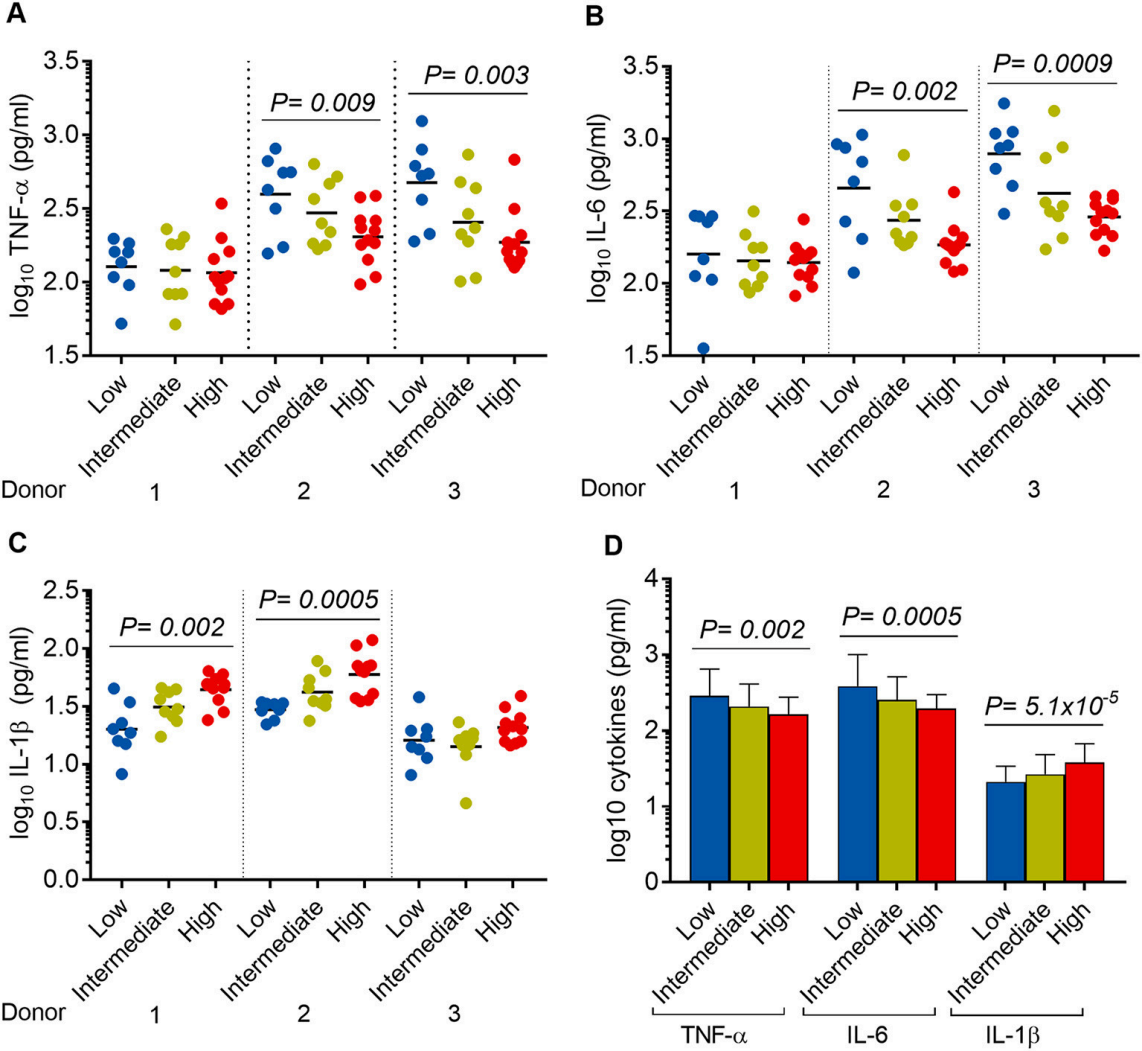
Association between *Mtb* virulence and cytokine responses in infected hMDM. hMDM from three independent donors were infected with 28 *Mtb* isolates and lab strain H37Rv. Supernatants were harvested after 24 h infection. **(A)** TNF-α, **(B)** IL-6, and **(C)** IL-1β expression for hMDM infected with low (*n* = 8), intermediate (*n* = 9), or high (*n* = 12) virulence *Mtb*. Experiments were performed in triplicate and mean values presented. Bars in plots represent median values. **(D)** Average cytokine responses for infected hMDM from three donors. Error bars represent mean values ± SD. Statistical comparisons were made using a linear trend test across the three virulence phenotypes.

It was noted that 8/29, 9/29, and 12/29 isolates showed low, intermediate, and high virulence phenotype in hMDM, respectively (Figure 5). Linear trend tests showed significant associations between virulence phenotype and concentrations of TNF-α, IL-6, and IL-1β in 2/3 donors (Figure 7). Increased virulence was associated with a reduced level of TNF-α [linear trend test, *P* = 0.009 (donor 2), *P* = 0.003 (donor 3)] (Figure 7A), and IL-6 [linear trend test, *P* = 0.002 (donor 2), *P* = 0.0009 (donor 3)] (Figure 7B), but intriguingly, with an increased level of IL-1β [linear trend test, *P* = 0.002 (donor 1), *P* = 0.0005 (donor 2)] (Figure 7C). Combined cytokine data from three donors showed significantly decreased concentrations of TNF-α and IL-6 but increased concentration of IL-1β in more virulent isolates (Figure 7D).

## Discussion

Mycobacterial virulence may be an important factor influencing heterogeneity in tuberculosis clinical manifestations and treatment responses. Tissue damage is likely to be involved in at least two stages of *Mtb* pathogenesis: liquefaction of granulomas preceding reactivation of latent infection and lung cavitation associated with advanced disease (Dheda et al., 2005); thus ability to lyse host cells could be a potential virulence-associated phenotype of *Mtb*. In fact, previous studies have shown virulent *Mtb* tend to induce necrotic cell death of infected macrophages with lysis of cytoplasmic membranes and release of intracellular constituents including the bacilli (McDonough and Kress, 1995; Dobos et al., 2000; Park et al., 2006). Interestingly, Sohn et al. even suggest that necrosis of infected macrophages, but not the intracellular growth of *Mtb* can reflect their virulence (Sohn et al., 2009). However, how the *in vitro* virulence links to TB clinical pathogenesis and transmission has not been clear.

Therefore, we assessed *Mtb* virulence in a large number of clinical isolates from pulmonary TB patients by macrophage lysis and correlated with multiple factors, ranging from *in vitro* macrophage infection to the bacterial load in patient's sputum. Such analysis was able to reveal a link between bacterial virulence, TB pathogenesis and transmission. First, we observed the lysis of THP-1 cells infected with 153 *Mtb* clinical isolates varied, ranging from no or very few cells lysed to 100% cells lysed after 6 days of infection. Such outcomes mirrored the diversity in the bacterial virulence, which spans a spectrum from low to high. High virulence strains showed tenfold higher host cell lysis than low virulence isolates in both THP-1 and hMDM, showing that high virulence bacteria have distinct ability to induce macrophage lysis.

Further, we measured bacterial growth and the expression of TNF-α and IL-6 in infected macrophages. Our results confirm previous findings that highly virulent isolates have the ability to modulate the killing of macrophages (Wong et al., 2007) and the proinflammatory response (Manca et al., 2001; Theus et al., 2005) to achieve better survival and growth. When reaching a threshold intracellular number, virulent *Mtb* lysed macrophages to escape for spreading infection (Lee et al., 2006). Consistent with the *in vitro* findings, we also found the first association of mycobacterial burden in patients' sputum with virulence phenotypes measured by host cell lysis. The explanation for more virulent strains having higher bacterial loads in sputum could be that these bacteria have increased ability to lyse macrophages in granulomas to form caseous necrosis, which may provide an environment for rapid bacterial proliferation (Ramakrishnan, 2012). Previous studies have shown an association between high bacterial load with cavities and multi-drug resistance in pulmonary TB patients (Palaci et al., 2007; Sander et al., 2016). Associations between *Mtb* virulence measured by host cell lysis and lung damage and treatment response should be further studied.

While the levels of TNF-α and IL-6 were reduced in hMDM infected with highly virulent *Mtb*, we observed an increased level of IL-1β in this model. Previous studies have demonstrated the consistently reduced levels of these three proinflammatory cytokines produced by hMDM in response to virulent *Mtb* (Reiling et al., 2013; Chen et al., 2014). However, several studies have shown the differential expression of TNF-α, IL-6, and IL-1β (Novikov et al., 2011; van Laarhoven et al., 2013). In a PBMC model, modern Beijing strains induced 2-fold less IL-1β than ancient Beijing strains, with no significant difference in the levels of TNF-α and IL-6 (van Laarhoven et al., 2013). The data support the hypothesis that the expression of IL-1β is regulated independently from other proinflammatory cytokines and may be manipulated by different *Mtb* strains (Portevin et al., 2011; Krishnan et al., 2013).

In our study, we observed an increased level of IL-1β associated with highly virulent isolates. There are different signaling pathways of IL-1β induction in macrophages. Increased production of IL-1β in macrophages infected with *Mtb* is dependent on caspase-1 and serine proteases as they may cleave pro-IL-1β to active form, rather than due to alteration in IL-1β transcription or levels of pro-IL-1β (Krishnan et al., 2013). Therefore, this potential mechanism for the increased production of IL-1β in highly virulent isolates shown in our experiments needs further study.

In addition, we observed strong association of high virulence phenotype with East Asian/Beijing lineage. There are many studies also indicating that *Mtb* lineage may influence virulence. Different models of infection with East Asian/Beijing genotype have suggested that this genotype is more virulent and less proinflammatory than other strains (Reiling et al., 2013; Via et al., 2013). The East Asian/Beijing lineage may also be more likely to progress to active TB and transmit infection to others (Holt et al., 2018). We found East Asian/Beijing isolates were associated with rapid cell lysis in macrophage models, which may serve to facilitate release and dissemination of mycobacteria *in vivo*. This was further supported by the observation that there was an increased bacterial number in sputum of patients infected with virulent *Mtb* defined by rapid macrophage lysis, as bacterial load in sputum is one of the risk factors for TB transmission. A recent study has suggested that different *Mtb* lineages may carry their unique lineage-specific genetic markers in virulence genes (Koster et al., 2018). Therefore, the association of *Mtb* genetics and the virulence defined by macrophage lysis will be investigated in the next stage of this work.

Overall, our research revealed a wide variation in bacterial virulence measured by macrophage lysis among *Mtb* clinical isolates. We also investigated the influence of strain virulence in clinical context and on host-pathogen interactions in macrophage models. We found that virulence is associated with bacterial burden in sputum from patients, bacterial lineage, bacterial survival, and cytokine responses in macrophages. Our findings suggest that the success of virulent East Asia/Beijing strains in disease establishment and transmission could be connected to their ability to survive and grow in macrophages, a characteristic that may be promoted by reduced TNF-α and IL-6 expression, and increased IL-1β. Further studies on association between *Mtb* virulence and clinical consequences and underlying genetic mechanism of lineage-specific virulence are needed.

## Author contributions

TT, NT, NP, and GT conceived and designed the experiments. TT, HN, SV, HH, DT, VH, TD, and NH did the experiments and collected the data. TT, HN, SV, PA, GT, and NT analyzed and interpreted the data. TT, HN, SV, HH, DT, VH, TD, PA, NP, NH, GT, and NT drafted, revised the manuscript, and approved the final version.

### Conflict of interest statement

The authors declare that the research was conducted in the absence of any commercial or financial relationships that could be construed as a potential conflict of interest.
