# Supplementary material for: Virulence of Mycobacterium tuberculosis Clinical Isolates Is Associated With Sputum Pre-treatment Bacterial Load, Lineage, Survival in Macrophages, and Cytokine Response
Source: Front Cell Infect Microbiol. 2018 Nov 27;8:417. doi: 10.3389/fcimb.2018.00417 (PMC6277702; doi:10.3389/fcimb.2018.00417)
Supplement: Supplementary file 1 [file Table_1.pdf]

*Supplementary Material*

# Virulence Of *Mycobacterium Tuberculosis* Clinical Isolates Is Associated With Sputum Pre-treatment Bacterial Load, Lineage, Survival In Macrophages, And Cytokine Response

**Trinh T. B. Tram, Hoang N. Nhung, Srinivasan Vijay, Hoang T. Hai, Do D. A. Thu, Vu T. N. Ha, Tran D. Dinh, Philip M. Ashton, Nguyen T. Hanh, Nguyen H. Phu, Guy E. Thwaites, Nguyen T. T. Thuong**

*** Correspondence:** Nguyen T. T. Thuong, thuongntt@oucru.org

## 1 Supplementary Tables

**Supplementary Table 1**. **Percentage of cell lysis of THP-1 infected with 153 *Mtb* clinical isolates**

| Cell lysis (%) / Day (N, %) | 0 - 5 | ≤5 - 10 | ≤10 - 30 | ≤30 - 50 | ≤50 - 70 | ≤70 - 90 | ≤90 - 100 |
|---|---|---|---|---|---|---|---|
| **1** | 153 (100.0) | 0 (0.0) | 0 (0.0) | 0 (0.0) | 0 (0.0) | 0 (0.0) | 0 (0.0) |
| **2** | 151 (98.7) | 2 (1.3) | 0 (0.0) | 0 (0.0) | 0 (0.0) | 0 (0.0) | 0 (0.0) |
| **3** | 127 (83.0) | 9 (5.9) | 12 (7.8) | 5 (3.3) | 0 (0.0) | 0 (0.0) | 0 (0.0) |
| **4** | 80 (52.3) | 9 (5.9) | 30 (19.6) | 14 (9.2) | 8 (5.2) | 4 (2.6) | 8 (5.2) |
| **5** | 61 (39.9) | 4 (2.6) | 17 (11.1) | 19 (12.4) | 15 (9.8) | 10 (6.5) | 27 (17.6) |
| **6** | 34 (22.2) | 3 (2.1) | 20 (13.1) | 15 (9.8) | 27 (17.6) | 8 (5.2) | 46 (30.1) |

N: number of isolates
